# Expanding the Ontology of Organizational Structures of Trauma Centers and Trauma Systems

**Published:** 2024-07

**Authors:** Diya Mehta, Justin M. Whorton, Reza Shahriari, Eric D Ragan, Jonathan P. Bona, William R. Hogan, Kevin W. Sexton, Mathias Brochhausen

**Affiliations:** 1 Harvey Mudd College, 301 Platt Blvd, Claremont, CA 91711, USA; 2 University of Arkansas for Medical Sciences, 4301 W Markham St, Little Rock, AR 72205, USA; 3 University of Florida, 201 Criser Hall PO Box 114000 Gainesville, FL 32611, USA; 4 Medical College of Wisconsin, 8701 W Watertown Plank Rd, Milwaukee, WI 53226, USA

**Keywords:** medical ontologies, trauma centers, organizational structures, patient outcomes

## Abstract

A knowledge gap exists regarding the impact of organizational parameters of trauma centers and patient outcomes. This is partially due to such organizational parameters being understudied. The Ontology of Organizational Structures of Trauma Centers and Trauma Systems (OOSTT) provides a controlled vocabulary to study that specific area. It is used in tools created by the TIPTOE project to provide trauma stakeholders with novel insights on role of organizational parameters and patient outcomes. This paper reports the extension of OOSTT to cover relevant patient outcome measures.

## 1. Introduction

In the United States in 2020, trauma is the leading cause of death for individuals under the age of 45 [1]. Despite growing standardization of clinical trauma care, at Level 1 (L1) and Level 2 (L2) trauma centers, there remains significant variability in patient outcomes across trauma centers on both levels [2, 3]. We hypothesize that this variability in patient outcomes is partially created by variability in organizational parameters of the trauma centers, which is an understudied subject. By organizational parameters we mean parameters of a trauma care environment, e.g., a trauma center, that describe how the care, documentation of care, and quality improvement measures are organize. The organizational parameters include, but are not restricted to key roles, e.g. trauma medical director, trauma program manager and trauma registrar, the obligations and privileges associated with those roles, the staffing of the trauma team, including credentials of participating providers, the availability of medical specialities and sub-specialties in or to the trauma team. The Ontology of Organizational Structures of Trauma Centers and Trauma Systems (OOSTT) is aimed to help address the knowledge gap regarding organizational structures. Its initial releases cover representation of trauma centers and trauma systems, their components, and the roles of professional and deontic roles that are part of these organizations [4]. OOSTT has been tested and validated to provide a controlled vocabulary for trauma centers and trauma systems organization [5]. It has been used to collect organizational data of trauma centers and trauma systems for the Comparative Assessment Framework of Environments of Trauma Care (CAFE) web service [6]. OOSTT is an OBO Foundry ontology, that is open access and can be used by developers and other ontologies to represent medical roles (e.g., trauma medical director), and organizational units (e.g., trauma team), and core components of trauma care (e.g., trauma centers).

In 2022, the second phase of the CAFE project started, and was renamed Trauma Institutional Priorities and Teams for Outcomes Efficacy (TIPTOE). The purpose of this phase is the evolution of trauma center quality improvement fostering adding scientific evidence regarding impact of organizational parameters on patient outcomes in L1 and L2 trauma centers. TIPTOE is recruiting 230 L1 and L2 trauma centers to fill in the survey about organizational parameters, similar to the CAFE web service [8], and provide their Trauma Quality Improvement Program (TQIP) data. TQIP is an initiative by the American College of Surgeons, Committee on Trauma aimed to improve the quality of care for trauma patients [7]. It collects data from trauma centers and provides feedback about performance and identifies improvements to be implemented by trauma center staff to improve outcomes [7].

**Figure 1:**
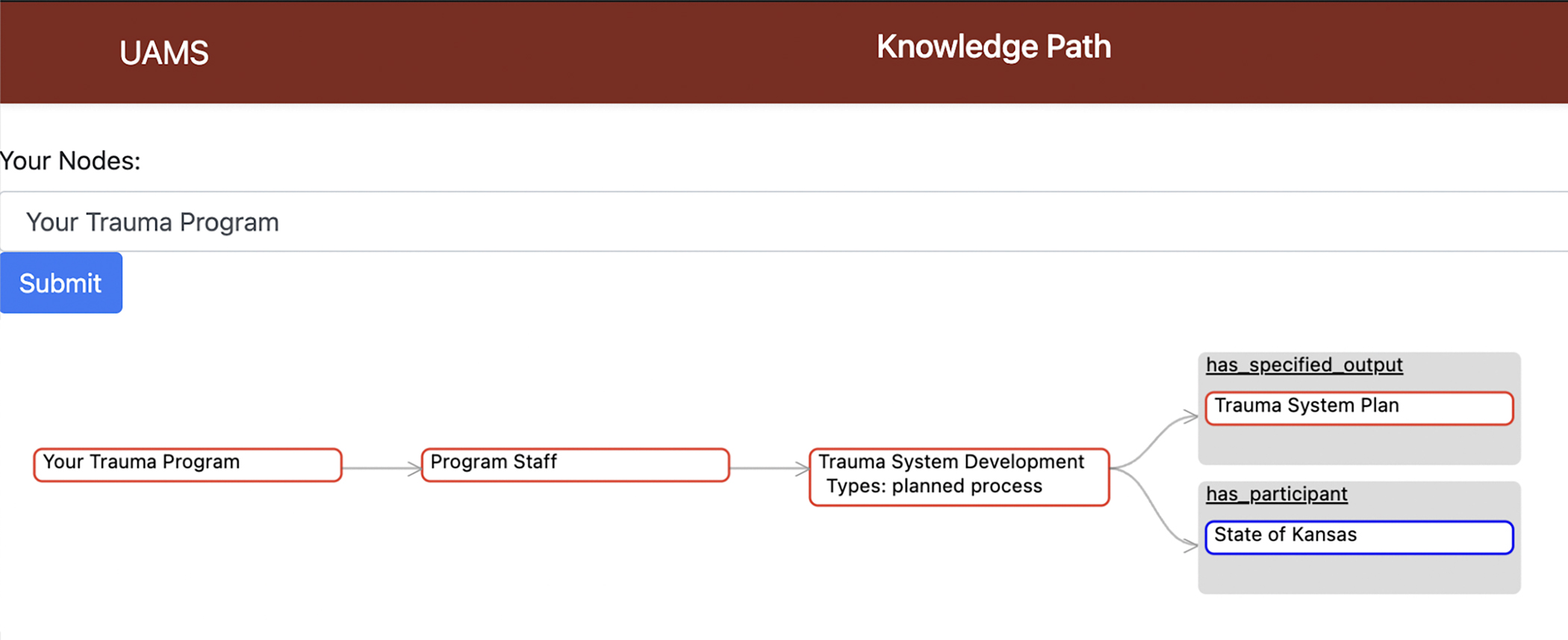
Screenshot of the TIPTOE Knowledge Path Explorer. Showing an example how a user can explore the knowledge graph about their trauma program.

One tool TIPTOE has developed is the Knowledge Path Explorer (KPE), that allows trauma center stakeholders to explore a knowledge graph that links organizational parameters of their institution to patient outcomes. The KPE graph is organized using OOSTT. Figure 1 shows the design of the KPE pilot that we are currently reviewing with medical staff for enhancements to design and functionality. The current visual graph interface allows inspection of specific parameters of interest while also providing the added benefit of showing context of the ontological information and relationships to other related parameters. Through participatory design with medical stakeholders and center leadership, the system will evolve to accommodate a broad range of data exploration goals. In this paper, we report the extensions of OOSTT, which are necessary to cover patient outcome data; something that was not necessary to the first phase of the project. We also present early results on how the OOSTT extension allows exploring TIPTOE data regarding two core competency questions:

1. How does the number of general surgeons with Advanced Trauma Life Support (ATLS) certification at your trauma center affect the number of major complications including death?
2. How does the inter-correlation between neurosurgeons taking call exclusively and the number of neurosurgeons with certified 18 hours of trauma-specific Continued Medical Education (CME) affect length of stay?

## 2. Methods

### 2.1. OBO Foundry and OBO Ontologies

The Open Biological and Biomedical Ontology Foundry (OBO Foundry) (http://obofoundry.org/) is a library of open source, community developed biological and biomedical ontologies agreeing to a set of overarching principles [8, 9]. The OBO Foundry aims at “facilitating the development, harmonization, application and sharing of ontologies (...)”[9]. The following OBO Foundry ontologies were used to expand OOSTT:

The Ontology for Modeling and Representation of Social Entities (OMRSE), initially names Ontology for Medically Related Social Entities, is ”a realist representation of medically related social entities”[10]. The scope has been expanded to cover ”that various entities that arise from human social interactions, such as social acts, social roles, social groups, and organizations”[11].

The MONDO Disease Ontology ”provides a sustainable and fully-provenanced approach to integrating disease concepts from numerous sources across disease categories”[12]. It currently represents over 22.000 disease concepts that represent 90.000 source concept from 17 disease resources[12].

The Infectious Disease Ontology (IDO) represents ”entities generally relevant to both the biomedical and clinical aspects of infectious diseases, including terms such as pathogen, host, vector, and vaccine”[13].

The Medical Action Ontology (MAxO) is ”a comprehensive open source computational representation of medical diagnostics, preventions, procedures, interventions, and therapies”[14]. MAxO currently contains more than 1.700 terms representing medical actions, such as medical procedures, interventions, therapies, and measurements[14].

### 2.2. OOSTT

OOSTT is a publicly available ontology that is part of the OBO Foundry and follows OBO Foundry principles. OOSTT can be accessed via http://purl.obolibrary.org/obo/oostt.owl. Additional information and tools, e.g., an issue tracker, can be found at OOSTT’s git repository: http://github.com/OOSTT/OOSTT. OOSTT uses Basic Formal Ontology [15]as its top level ontology and covers the domain of trauma center and trauma system organizational parameters. In 2022, the design principles and coverage of OOSTT have been reviewed by WRH, who was not involved in the initial OOSTT development. The adjustment and changes suggested by that review have been implemented during 2023.

### 2.3. OOSTT Extensions

This current ontology development step aims to provide ontological representation for TQIP data elements to enable the integration of TQIP data with data on organizational structures in the TIPTOE project and, specifically in the KPE. This extension was done using two different approaches: a) terminology-driven to broaden OOSTT coverage, b) data-driven providing representation for the 3 patient outcomes TIPTOE focuses on.

#### 2.3.1. Terminology-driven Extension

To foster integration with trauma outcome data nationwide, the study started with definitions and labels from TQIP’s data dictionary, the National Trauma Data Standard (NTDS)[16]. ”The NTDS Dictionary is designed to establish a national standard for the exchange of trauma registry data, and to serve as the operational definitions for the National Trauma Data Bank (NTDB)”[16]. It is a crucial component of TQIP, since the standardization provided by the NTDS allowed the addition of assessment of patient outcomes to the trauma center verification process[17]. Our project was done as a Summer Research Internship by DM. First, 20 terms from the NTDS were identified for implementation in the Web Ontology Language (OWL) and inclusion in OOSTT. All 20 terms are listed in the first column of Table 1. These terms were picked based on priority regarding project needs. A spreadsheet was created to account for changes made to each term and its curation status.

First, the label of each term from NTSD was reviewed for consistency with the label format suggested by [18]. Each label that did not follow the format was edited to their singular form, the expanded version of abbreviations/symbols, and in lowercase lettering. For instance, “ICD-10 INJURY DIAGNOSIS” became “international classification of diseases tenth revision injury diagnosis”. Additionally, all acronyms were expanded, to prevent misunderstandings, following the OBO Foundry principle on naming[19].

There was one instance where one NTDS term, required three proposed OOSTT terms, to specifically represent the NTDS term’s specified values: According to the NTDS database, the term “alternative home residence” represents individuals that are either homeless, living at a temporary residence, or are undocumented. Since those three values represent situations that are not easily represented by one superclass, we decided to discard “alternative home residence”. It was replaced by three terms capturing its respective values: “homeless”, “temporary address”, and “undocumented immigrant”. By following these guidelines, we are preventing incorrect hierarchical structures, such as claiming that an instance of an undocumented immigrant is also a member of the class ‘alternative home residence’, once we build the OWL hierarchy. This complies with the requirement to build taxonomies on the basis that every member of the child class is also a member of the parent class[20].

Second, we reviewed the definitions of the NTDS terms and found that some of them are defined in a circular manner, viz. the label or parts thereof are used as the definition. For instance, NTDS defines the term ICD-10 INJURY DIAGNOSIS as “diagnosis related to all identified injuries”. The definition does not explicate what the words ”diagnosis” and ”injury” actually mean. Additionally, the phrase ICD-10 is addressed by the definition. We propose an alternative definition: “An information content entity that is about an injury borne by a patient and that expresses the diagnosis in an ICD 10 code”. This definition uses the next superclass (genus), ”information content entity”, and gives differentiating characteristics. Thus, the term is defined by it being a member of a specific superclass and its specific, defining characteristics, following the format suggested by OBO Foundry principles[21]. Each definition was rewritten following this format.

The revised and edited terms yielded 22 potential new ontology classes. Three of those terms, severe sepsis, unplanned intensive care unit admission, and unplanned endotracheal intubation, were pushed to the data-driven extension process for consistency. The other 19 terms were manually checked against OBO Foundry ontologies, to prevent duplication. No duplication with those 19 terms has been detected. Hence, they were implemented in OWL[22] using the Protege ontology editor [23]. A complete list of all 20 NTSD terms, the proposed OOSTT label, and the OWL implementation status can be found in Table 1. The implementation was done by manually creating a novel OWL file. This file imported BFO [15]and the Information Artifact Ontology (IAO)[24]. The development team (DM and MB) decided to import IAO, since 10 of the initial potential new OOSTT classes were subclass of ‘information content entity’ [24], which is not part of BFO, nor does BFO contain another class representing information. The resulting OWL file was merged with the latest release of OOSTT resulting in OOSTT release version 2024–01-25 (https://github.com/OOSTT/OOSTT/tree/2024-01-25).

#### 2.3.2. Data-driven Extension

In parallel to the terminology-driven approach, we needed to extend OOSTT to cover the 3 patient outcomes the TIPTOE project focuses on: mortality, length of stay, and major complications. For major complications the representation of clinical conditions and situations was also needed. To represent the various major complications, we required classes from MONDO[12], IDO[25], and MAxO[14] using a MIREOT[26] Protege Plugin. The details on the imported classes can be found in Table 2. To represent mortality using discharge disposition data and length of stay, we used MIREOT to import two classes from OMRSE[10] (see Table 2). The minimum information to reference an external ontology term (MIREOT) guidelines were initially created to develop the Ontology for Biomedical Investigation (OBI)[27]. MIREOT enables the reuse of pre-existing ontology resources, e.g., classes and object properties to avoid duplication[26]. MIREOT is ”independent of any design principle, and provides a mechanism by which external ontology terms can be selectively imported, even if they do not use a particular upper ontology(...)”[26]. This is particularly relevant for developing multiple ontologies in one unified environment, for instance, in the OBO Foundry[8, 9]. Hanna et al. implemented the MIREOT guidelines into a Protege plug in that allows to drag and drop classes and object properties from existing OBO Foundry ontologies in ontology project developed in Protege[28].

Examples of the classes proposed based on the NTDS are given in Table 1. In total, 36 classes were imported to OOSTT in this step. In addition, 8 classes were created newly in OOSTT: unplanned intensive care unit admission process, intensive care unit admission process, unplanned surgical procedure, unplanned endotracheal intubation, cardiopulmonary resuscitation, patient discharge disposition information, total intensive care unit length of stay data item. Three of these classes had also been identified in the terminology-driven extension approach.

## 3. Results

**Figure 2:**
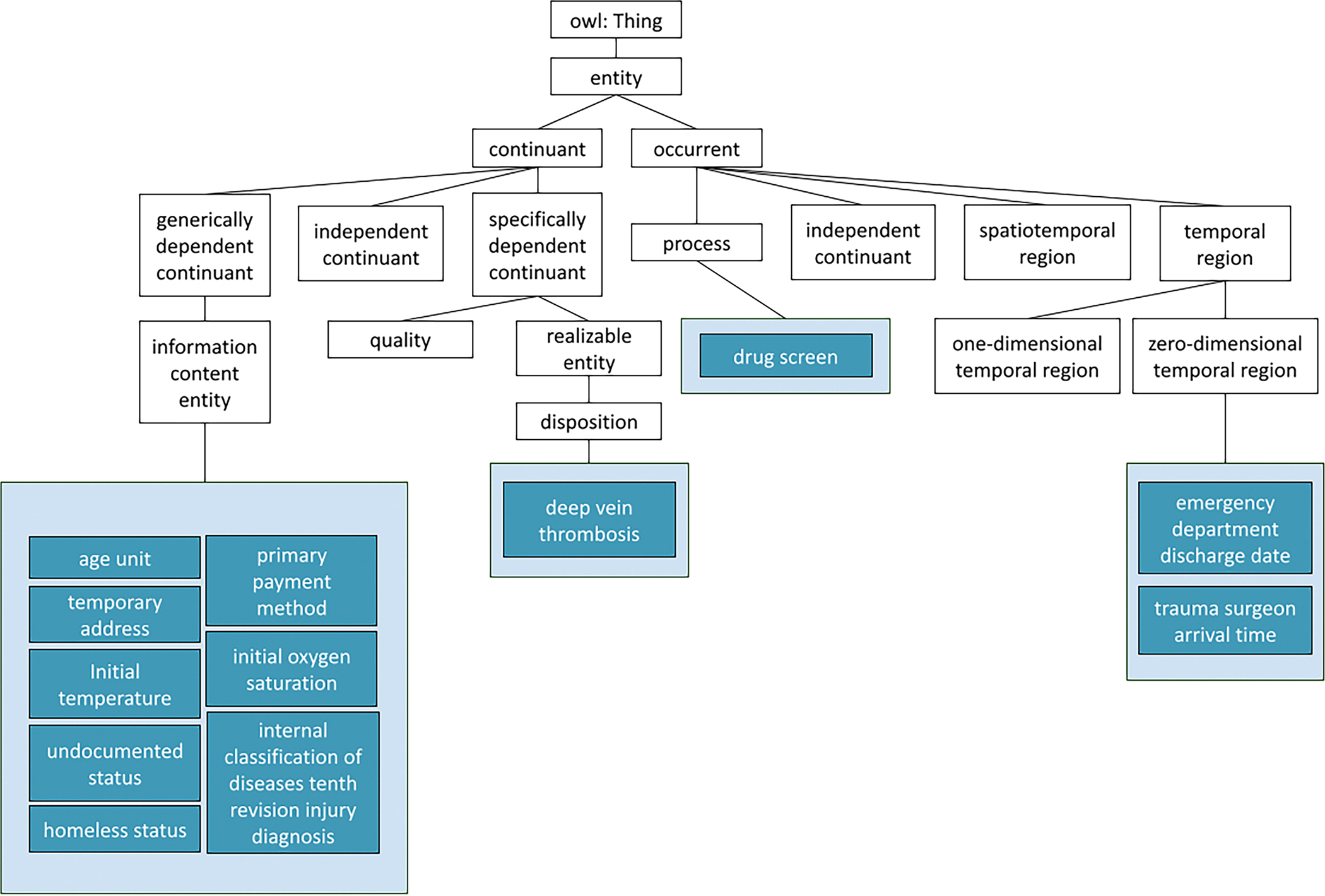
Visual representation of the created OWL file. BFO and IAO subclasses represented by white boxes and newly created NTDS terms categorized highlighted in blue.

All terms, their NTDS definitions, and the definitions revised in accordance with the principles and practices mentioned above can be found here: https://tinyurl.com/OOSTTe. Figure 2 shows how 11 terms implemented in OWL as part of the terminology-driven extension approach extend BFO. The definitions have been revised by KWS, our trauma surgery expert. In total, OOSTT was expanded by 55 classes; 36 imported classes and 19 new classes. The OOSTT release that includes all extensions discussed in this paper can be accessed at: http://purl.obolibrary.org/obo/oostt/release/2024-01-25/oostt.owl.

The ontology development described here makes these outcome patient measures available in the KPE. We are currently in the process of conducting a usability study of the KPE. Due to ongoing data collection and analysis, this utilizes with a virtual data set that includes instance data on patient outcomes. While the usability study is still ongoing and results from it are not yet available, in preparation we internally tested the functionality of the tool with the newly created OOSTT classes. We can report that the ontology extension described in this paper, allows successfully retrieving information on the following two topics from the TIPTOE triple store using the KPE:

1. How does the number of general surgeons with ATLS certification at your trauma center affect the number of major complications including death?
2. How does the intercorrelation between neurosurgeons taking call exclusively and the number of neurosurgeons with certified 18 hours of trauma-specific CME affect length of stay?

In addition to those two data exploration scenarios, the extension also enable multiple other scenarios related to patient outcomes and more detail on patient demographics, diagnostics, and treatment. Once the usability testing, consisting of two formative usability studies and one summative study at the end of the project are completed, the tool will be made available.

## 4. Discussion

In previous development of OOSTT, we added definitions for domain experts in addition to the genus-differentia definitions and we validated those definitions with domain experts. This step has not yet been undertaken with this extension, but we plan to address this issue in the next project year.

At this point, we have not yet conducted statistical analyses to assess which features of trauma centers affect patient outcomes. As the results of those statistical analyses become available, specifically the relationships between organizational features and patient outcomes those will be added to OOSTT, too.

## 5. Conclusions

The integration of extensions into the Ontology of Organizational Structures in Trauma Centers and Trauma Systems (OOSTT) significantly enhances our ability to discern the nuanced effects that organizational structures and parameters exert on patient outcomes. This methodological advancement facilitates a novel approach to examining the intricate relationships within healthcare delivery systems. With the new extension OOSTT covers not only the multispecialty composition of trauma care teams, but also key patient outcomes along the care pathway, such as admission, readmission, and discharge. In addition, we also capture diagnoses that represent unintended medical comlications, such as sever sepsis. By deploying this enhanced ontology, our representation can encompass both multispecialty and single clinical service outcomes. For instance, it enables a thorough examination of multispecialty outcomes, such as readmission rates to the Intensive Care Unit (ICU), alongside the analysis of outcomes for specific conditions, like the care pathway for isolated femur fractures. This dual perspective permits a comprehensive exploration of complex system dynamics, specifically focusing on their impact on clinical care. Through this lens, we gain a more profound understanding of the interplay between organizational structures and patient health results, providing valuable insights into potential areas for improvement.

## Figures and Tables

**Table 1:** OWL implementation status of new OOSTT terms based onNTDS terms.

NTSD term	OOSTT term	In OWL?

Age Units	age unit	Yes

ICD-10 Injury Diagnosis	international classification of disease tenth revision injury diagnosis	Yes

Drug Screen	drug screening	Yes

ED Discharge	emergency department discharge date	Yes

Initial ED/Hospital Oxygen Saturation	initial oxygen saturation	Yes

Initial ED/Hospital Temperature	initial temperature	Yes

Trauma Surgeon Arrival Time	trauma surgeon arrival time	Yes

Primary Method of Payment	primary payment method	Yes

Deep Vein Thrombosis (DVT)	deep vein thrombosis	Yes

Acute Kidney Injury (AKI)	acute kidney injury	No

Severe Sepsis	severe sepsis	Yes *!

Unplanned Admission To ICU	unplanned intensive care unit admission	Yes*

Unplanned Intubation	unplanned endotracheal intubation	Yes*

ICD-10 Hospital Procedures	international classification of diseases tenth revision hospital procedure	No

Incident Location Zip/Postal Code	incident zip/postal code	No

Patient’s Occupational Industry	patient’s occupation	No

Protective Devices	protective device	No

Total ICU Length Of Stay	intensive care unit length of stay data item	Yes

Advance Directive Limiting Care	advance directive limiting care	No

Alternate Home Residence	homeless status	Yes
	temporary address	Yes
	undocumented status	No

Terms marked * were implemented during data-driven extension; terms marked ! were imported from IDO.

**Table 2:** Examples of classes imported to OOSTT using MIREOT

Source Ontology	Terms

OMRSE	patient discharge, admission process
MONDO	respiratory failure, acute respiratory distress syndrome, myocardial infarction, cardiac arrest, pulmonary embolism, stroke disorder
IDO	severe sepsis
MAXO	endotracheal intubation
